# Decision-making factors for best supportive care alone and prognostic factors after best supportive care in non-small cell lung cancer patients

**DOI:** 10.1038/s41598-019-56431-w

**Published:** 2019-12-27

**Authors:** Haruna Kitazawa, Yuichiro Takeda, Go Naka, Haruhito Sugiyama

**Affiliations:** Department of Respiratory Medicine, National Center for Global Health and Medicine 1-21-1 Toyama, Shinjuku-ku, Tokyo 162-8655 Japan

**Keywords:** Non-small-cell lung cancer, Palliative care

## Abstract

Among patients with non-small cell lung cancer (NSCLC), best supportive care (BSC) is well-known to improve patient’s quality of life and prolong survival. This study aimed to clarify (1) the decision-making factors of BSC alone and (2) the prognostic factors after selection of no further anticancer therapies. We retrospectively reviewed the clinical data of patients with NSCLC between November 2004 and February 2014, who received BSC as only therapy and BSC after completion of anticancer therapies. One hundred eighteen patients received BSC alone. Among 860 patients treated with anticancer therapies, 236 were selected as control group, 160 of whom received BSC after anticancer therapy. The significant reasons for receiving BSC alone were: comorbidities of dementia, poor Eastern Cooperative Oncology Group performance status (ECOG-PS), patients’ wishes, pulmonary comorbidities, wild type epidermal growth factor receptor (EGFR), relevant social background and psychiatric comorbidities. Poor prognostic factors at the start of BSC were poor ECOG-PS, presence of disseminated intravascular coagulation (DIC), and history of anticancer therapy. NSCLC patients with comorbidities, wild type EGFR, and relevant social background factors tended to receive BSC alone. Post-cancer therapy NSCLC patients and those with DIC and declining ECOG-PS have a shorter survival period from the start of BSC.

## Introduction

Lung cancer is a leading cause of cancer-related deaths worldwide^1^. In spite of the recent development of anticancer therapies for metastatic non-small cell lung cancer (NSCLC), many patients still experience a poor prognosis^2^. Most patients with advanced cancer want to know the amount of time they have left^3^. Moreover, prediction of prognosis is essential when determining the appropriate anti-cancer therapy. A recent study showed that early palliative care leads to improvements in both quality of life (QOL) and survival^4^. Hence, comprehensive treatment for NSCLC should include palliative care. The treatment for NSCLC consists of four therapeutic modalities: surgery, radiotherapy, cancer chemotherapy, and palliative care. Best supportive care (BSC) consists of appropriate palliative care without any other anticancer therapies. For all patients diagnosed with lung cancer, both anticancer therapies and BSC are essential to improve the patient’s QOL and survival^4–6^. However, since most types of anticancer therapies are associated with severe adverse effects and long-term disabilities, some patients hesitate to receive anticancer therapies despite these being the medical recommendation. When cancer patients decline anticancer therapies, BSC is the only available option. However, only few previous reports have investigated the factors related to selection of BSC alone as therapy and the factors related to prognosis after the treatment decision of BSC for patients with NSCLC. The aim of this study was to examine the decision-making factors for BSC alone and the prognostic factors after the decision for treatment with BSC using comparisons between NSCLC patients who received BSC alone and those who received BSC after completion of anticancer therapy.

## Methods

### Study design and patients

**Figure 1:**
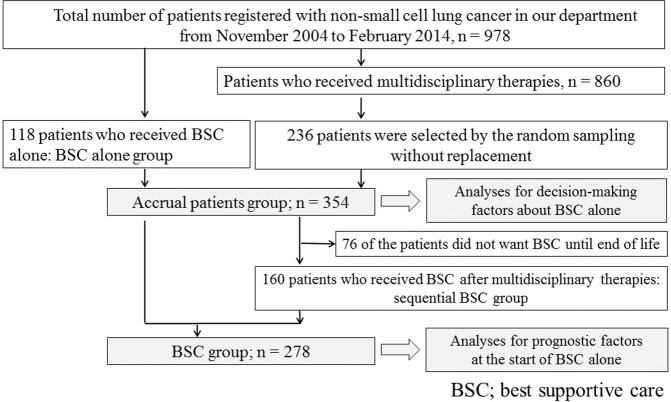
Flow chart for enrollment in this study.

In this retrospective study conducted in the Department of Respiratory Medicine of the National Center for Global Health and Medicine, all patients diagnosed with lung cancer were registered in the Lung Cancer database (Fig. 1). The study inclusion criteria were: (1) pathologically-confirmed diagnosis of NSCLC; (2) age 20 years or older; and (3) patients who received any kind of treatment for their lung cancer, both palliative and curative, or other anticancer therapies. From 2004 to 2014, after patients were diagnosed with NSCLC, they were offered multidisciplinary treatments (MTs) according to the guidelines published by the National Comprehensive Cancer Network (NCCN)^7^ or the Japanese Lung Cancer Society^8^ that were prevalent at that time. BSC was defined as “as early as possible care for the prevention or treatment of the symptoms of a disease, and the psychological, social and spiritual problems related to the disease, including palliative radiation or pharmacotherapy not for anti-cancer treatment but for relief from any symptoms”^9^. BSC alone was defined as “BSC alone in the absence of any other kind of MTs”. Patients were also included in the BSC alone group when they opted for BSC alone despite receiving explanation about the need for more definitive therapy according to the guidelines.

All patients in the lung cancer database of our department who were found to meet the inclusion criteria between November 2004 and February 2014 were included in the study. They were divided into the BSC alone group and MT group. We hypothesized that the decision-making of BSC would be associated with the patient’s desire for BSC. Thirty-nine of 118 (33%) patients in BSC group were willing to wish BSC. Our preliminary investigation indicated about 19% of patients in MT group finally wanted BSC. When the type I error was set at 0.05 (two-sided test) for the comparison of two groups and power was set at 80%, 234 of patients in MT group would be required for 117 of patients in BSC by the chi-squared test. We planned to enroll 118 patients for BSC alone and 236 for MT group. According to above consideration, the control group, which included twice the number of patients as the BSC alone group, was randomly sampled without replacement from among the patients who received MT. The BSC alone group and control group together constituted the “accrual group”. Logistic regression analyses were used to determine the decision-making factors for BSC alone in the accrual group. The BSC group comprised the BSC alone group and those patients who received BSC after completion of MT, excluding patients who did not want BSC until the end of life from the control group (sequential BSC group). Cox regression analysis was used to determine prognostic factors in the BSC group.

Data on the following variables were collected from each patient’s medical record and followed up until March 2015: patient characteristics, such as age, sex, comorbidities, and Eastern Cooperative Oncology Group performance status (ECOG-PS); social background, including the need for outside assistance or psychiatric hospitalization; oncologic information, such as histologic type, staging according to the 7th edition of the TNM Classification of Malignant Tumors^10^, gene mutation profile, in particular about epidermal growth factor receptor (EGFR) mutation; treatment information, such as anticancer therapy regimens, number of subsequent systemic therapies, history of palliative radiation, and the reason for selection of BSC; information on biochemical parameters, such as white blood cells (/µL), hemoglobin (g/dL), platelets (/µL), albumin (g/dL), aspartate aminotransferase (unit/L), alanine aminotransferase (unit/L), lactate dehydrogenase (unit/L), blood urea nitrogen (mg/dL), creatinine (mg/dL) and C-reactive protein (mg/dL).

### Ethical considerations

The study was conducted according to the principles of the Declaration of Helsinki. The study protocol was approved by certified review board of National Center for Global Health and Medicine (NCGM-G-002270-00). Informed consent from the patients for the use of their medical data in this retrospective study was obtained using the opt-out method and a disclosure document. The Japanese Ethical Guidelines for Medical and Health Research Involving Human Subjects, which are established by the Ministry of Education, Culture, Sports, Science and Technology and the Ministry of Health, Labour and Welfare, approves opt-out method as one of informed consent in case of retrospective cohorts without invasion and intervention^11^. According to the Japanese Ethical Guidelines for Medical and Health Research Involving Human Subjects, we made announcement about this study and obtained informed consent from research subjects by displaying the disclosure document in the hospital from the approval data by certified review board of National Center for Global Health and Medicine until 31st August 2018. We described in the disclosure document about the objectives, methods, time period, enrolling criteria, handling of personal information including process of anonymization, means to disclose information on research, and means to respond to the consultation made by the research subjects in the document.

### Statistical analysis

The primary outcome was to find the clinical factors related to the choice of BSC alone. Secondary outcomes were the prognostic factors in patients with NSCLC receiving BSC alone as compared to those in the sequential BSC group.

Fisher’s exact test or Mann-Whitney U test was used to analyze background characteristics of the accrual and BSC groups. We performed logistic regression analysis to investigate decision-making factors about BSC alone, and Cox regression analysis to investigate prognostic factors at the start of BSC, which was defined as the date of decision-making regarding BSC. Survival analysis, as an indication of prognosis, was performed using the Kaplan-Meier method and comparisons were made using the log-rank test. Overall survival (OS) was defined as the time from the date of pathological diagnosis to date of death due to any cause or date of last contact for subjects who were still alive. The survival duration following BSC was defined as the time from the beginning of BSC to death or last contact for living subjects.

Propensity score (PS) was calculated for the patient’s desire for BSC without anticancer therapy was constructed for each patient using a logistic regression model. We performed not only crude analysis, but also PS-adjusted analysis to reduce potential confounding and bias due to the retrospective nature of this study.

For model selection for multivariate analysis, we identified variables with a p-value less than 0.1 in univariate analysis. Spearman’s rank test and clinically clarified dependent variables were used to exclude the dependent variables from the above selected variables. A correlation coefficient (rho) of more than 0.3 as the absolute value with Spearman’s rank test indicated a significant association. Some models were constructed with only independent variables as candidates. Akaike’s Information Criterion (AIC) was used to select the best model among candidate models. In the final multivariate analysis using the simultaneous method, statistical significance was determined at a p-value of <0.05 with a two-sided test. All analyses were performed using SPSS Statistics software version 22 (IBM, New York, USA).

## Results

### Patients

A total of 978 patients were newly diagnosed with NSCLC between November 2004 and February 2014 (Fig. 1). Among them, 118 patients received BSC alone. As the control group, 236 patients who received MTs, i.e. twice as many as those who received BSC alone, were chosen from the remaining 860 patients by random sampling without replacement. Finally, a total of 354 patients were enrolled in this retrospective cohort as the “accrual group”.

### Decision-making factors about BSC alone

These were determined using the entire accrual group. The background characteristics of the accrual group are summarized in Supplementary Table S1 and the information about the treatment is shown in Supplementary Tables S3 and S4. The number of males was large in both groups, although there were no significant differences between the groups (p = 0.19). Patients in the BSC alone group were significantly older (p < 0.0001) and had more advanced cancer (p < 0.0001) and poorer ECOG-PS (p < 0.0001) than those who received MTs with supportive care group. Patients who received MTs with supportive care had more adenocarcinoma (p < 0.0001) or harbored more EGFR mutations (p < 0.0001) than those in the BSC alone group. In terms of comorbidities, patients in the BSC alone group had a greater incidence of pulmonary disease (p = 0.002), aspiration (p < 0.0001), dementia (p < 0.0001), renal dysfunction (p = 0.01), psychiatric disorders (p = 0.019), hematological disorders (p = 0.019), and relevant social background, such as the need for public assistance and psychiatric hospitalization (p = 0.009) than those in the MT group.

In order to know how patients with NSCLC made a decision about BSC alone, we conducted logistic regression analysis of the accrual group (Fig. 1). The results of crude logistic regression analysis are shown in Table 1. There were 29 variables with p-values less than 0.1 in univariate analyses. We constructed 12 sets of multivariate models composed of variables that were not correlated with each other. The best model selected by AIC from among the 12 models is shown in Table 1. This best model had 12 variables. Although 11 of the variables had statistically significant differences, 10 variables with odds ratios (OR) less than 0.85 or more than 1.18 were considered to be clinically useful^12^. Crude multivariate analysis indicated that the significant factors related to selection of BSC alone were: comorbidities of dementia, poor ECOG-PS, patients’ desire for BSC, wild type EGFR, comorbidities of pulmonary disease, high serum hemoglobin concentration, renal dysfunction, psychiatric disorders and hematological disorders, and presence of the relevant social background.

**Table 1:** Crude logistic regression analysis of choice of treatment with best supportive care alone.

Variable	Univariate Analyses			Multivariate Analysis		
	OR	95% CI	p-value	OR	95% CI	p-value
Age (low → high)	1.09	1.06–1.12	0.0001	1.11	1.06–1.17	**0.0001**
Dementia (absent vs. present)	18.9	5.53–62.5	0.0001	**45.5**	**7.35**–**250.0**	**0.0001**
Performance status (0 → 4)	3.75	2.82–5.00	0.0001	**6.76**	**4.17**–**11.0**	**0.0001**
Patient’s desire for best supportive care alone (absent vs. present)	5.03	2.99–8.48	0.0001	**22.7**	**7.94**–**66.7**	**0.0001**
Epidermal growth factor receptor mutation (mutant vs. wild type)	2.55	1.76–3.70	0.0001	**3.38**	**1.62**–**7.04**	**0.001**
Pulmonary disease (absent vs. present)	2.10	1.33–3.29	0.001	**3.95**	**1.66**–**9.43**	**0.002**
Hemoglobin (low → high)	0.91	0.81–1.01	0.08	**1.34**	**1.09**–**1.66**	**0.007**
Renal dysfunction (absent vs. present)	2.47	1.25–4.88	0.009	**5.35**	**1.49**–**19.2**	**0.01**
Social background (absent vs. present)	2.98	1.32–6.71	0.008	**6.25**	**1.30**–**30.3**	**0.022**
Psychiatric disorder (absent vs. present)	3.82	1.25–11.6	0.019	**12.1**	**1.43** –**100.0**	**0.022**
Blood disease (absent vs. present)	6.25	1.25–31.3	0.026	**10.1**	**1.02**–**100.0**	**0.048**
Aspartic acid aminotransferase (low → high)	1.01	1.00–1.03	0.06	1.01	0.99–1.03	0.45
Histologic type (adenocarcinoma vs. others)	2.71	1.70–4.31	0.0001		NI	
Stage (1 → 4)	1.31	1.14–1.51	0.0001		NI	
Albumin (low → high)	0.54	0.39–0.76	0.0001		NI	
Lactate dehydrogenase (low → high)	62.5	9.62–500.0	0.0001		NI	
C-reactive protein (low → high)	1.16	1.10–1.23	0.0001		NI	
White blood cell (×10^3^) (low → high)	1.12	1.06–1.18	0.0001		NI	

Abbreviations: OR, Odds ratio; CI, confidence interval; Social background includes a history of outside assistance and psychiatric hospitalization.

Variables with a p-value < 0.10 on univariate analysis were entered into multivariate logistical analysis by a simultaneous method.

NI, not included in the best multivariate logistic regression model.

The results of PS-adjusted logistic regression analysis are shown in Table 2. PS-adjusted univariate analyses indicated 26 variables with p-values less than 0.1. The best model selected from among 20 sets of PS-adjusted multivariate models also had 12 variables. PS-adjusted multivariate logistic regression analysis was performed using this best model, which indicated the following factors as being clinically significant in decision-making regarding BSC alone: dementia as a comorbidity (OR, 37.0; 95% confidence interval [CI], 6.54 to 200.0; p = 0.0001), poor ECOG-PS (OR, 6.10; 95% CI, 3.83 to 9.71; p = 0.0001), patients’ desire for BSC (OR, 22.2; 95% CI, 6.71 to 76.9; p = 0.0001), pulmonary disease as a comorbidity (OR, 3.83; 95% CI, 1.64 to 8.93; p = 0.002), wild type EGFR (OR, 2.68; 95% CI, 1.29 to 5.56; p = 0.008), presence of the relevant social background (OR, 5.44; 95% CI, 1.22 to 24.4; p = 0.027), and psychiatric disorder as a comorbidity (OR, 9.09; 95% CI, 1.26 to 66.7; p = 0.029) (Table 2).

**Table 2:** Propensity score-adjusted logistic regression analysis of choice of treatment with best supportive care alone.

Variable	Univariate Analysis			Multivariate Analysis		
	OR	95% CI	p-value	OR	95% CI	p-value
Age (low → high)	1.08	1.05–1.11	0.0001	1.11	1.05–1.16	**0.0001**
Dementia (absent vs. present)	20.4	5.85–71.4	0.0001	**37.0**	**6.54** –**200.0**	**0.0001**
Performance status (0 → 4)	3.77	2.83–5.03	0.0001	**6.10**	**3.83**–**9.71**	**0.0001**
Patient’s desire for best supportive care alone (absent vs. present)	4.02	2.22–7.30	0.0001	**22.2**	**6.71**–**76.9**	**0.0001**
Pulmonary disease (absent vs. present)	2.13	1.34–3.39	0.001	**3.83**	**1.64**–**8.93**	**0.002**
Epidermal growth factor receptor mutation (mutant vs. wild type)	2.97	2.00–4.42	0.0001	**2.68**	**1.29**–**5.56**	**0.008**
Social background (absent vs. present)	3.39	1.46–7.81	0.004	**5.44**	**1.22**–**24.4**	**0.027**
Psychiatric disorder (absent vs. present)	4.65	1.49–14.5	0.008	**9.09**	**1.26**–**66.7**	**0.029**
Renal dysfunction (absent vs. present)	2.52	1.25–5.08	0.010	3.18	0.92–11.0	0.068
Blood disease (absent vs. present)	6.67	1.27–34.5	0.025	6.37	0.69–58.8	0.103
Aspartic acid aminotransferase (low → high)	1.02	1.002–1.03	0.031	1.01	0.99–1.03	0.366
Sex (female → male)	1.57	0.94–2.64	0.086	1.36	0.51–3.64	0.534
Histologic type (adenocarcinoma vs. others)	2.94	1.81–4.79	0.0001		NI	
Albumin (low → high)	0.53	0.38–0.75	0.0001		NI	
Lactate dehydrogenase (×10^3^) (low → high)	83.3	12.4–500.0	0.0001		NI	
Doctor’s recommendation about best supportive care (absent vs. present)	32.3	10.3–100.0	0.0001		NI	
C-reactive protein (low → high)	1.16	1.10–1.23	0.0001		NI	
White blood cells (×10^3^) (low → high)	1.11	1.05–1.17	0.0001		NI	
Stage (1 → 4)	1.27	1.10–1.46	0.001		NI	

Social background includes the patient’s history of public assistance and psychiatric hospitalization.

Abbreviations: OR, Odds ratio; C.I., confidence interval; Variables with a p-value < 0.10 on univariate analysis were entered into the multivariate logistic analysis using the.

simultaneous method. NI, not included in the best multivariate logistic regression model.

### Survival from the beginning of BSC

The BSC group consisted of 278 patients, including 118 patients in the BSC alone group and 160 in the sequential BSC group. The remaining 76 patients in the control group were not included because they declined BSC. The background characteristics of the BSC group are summarized in Supplementary Table S2. Patients in the BSC alone group were significantly older (p < 0.0001) and had a better ECOG-PS (p < 0.0001) than those in the sequential BSC group. There were no significant differences between the two groups in terms of sex (p = 0.50). Patients in the sequential BSC group had more advanced disease at the start of BSC than those in the BSC alone group (p < 0.0001). In terms of comorbidities, a larger number of patients in the BSC alone group had dementia (p < 0.0001) and social issues (p = 0.01) as compared to the sequential BSC group. A larger number of patients in the sequential BSC group had pulmonary disease (p = 0.004) and liver dysfunction (p = 0.03) as compared to the BSC alone group.

**Figure 2:**
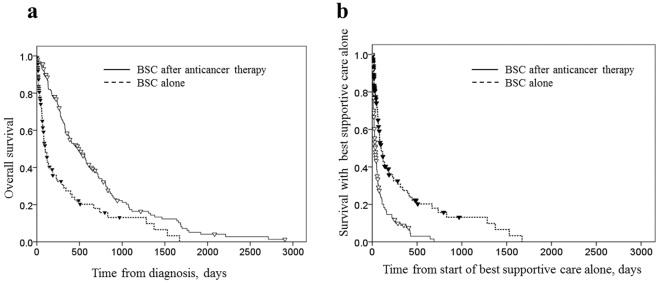
(**a**) Overall survival in the best supportive care group. Overall survival (OS) was calculated from the date of diagnosis of non-small cell lung cancer to the date of death among patients who received either best supportive care (BSC) alone or BSC after anticancer therapy. Median estimates of OS were as follows: 98.0 days (95% confidence interval [CI], 62.1 to 133.9 days) in the BSC alone group (118 patients, dotted line), and 492.0 days (95% CI, 349.2 to 634.8 days) in the BSC after anticancer therapy group (160 patients, solid line) (p < 0.0001 by the log-rank test). (**b**) Survival from the start of best supportive care alone in the best supportive care group. Survival was calculated as the duration from the date of diagnosis of non-small cell lung cancer in the best supportive care (BSC) alone group or the date of termination of anticancer therapy in the BSC after anticancer therapy group to the date of death. Median estimates of survival from the start of BSC alone were as follows: 98.0 days (95% CI, 62.1 to 133.9 days) in the BSC alone group (118 patients, dotted line), and 36.0 days (95% CI, 26.7 to 45.3 days) in the BSC after anticancer therapy group (160 patients, solid line) (p < 0.0001 by the log-rank test).

Median estimates of OS were significantly longer in the sequential BSC group than in the BSC alone group (492.0 days; 95% CI, 349.2 to 634.8 days vs. 98.0 days; 95% CI, 62.1 to 133.9 days; p < 0.0001) (Fig. 2a). However, survival from the start of BSC alone (the period that patients were treated by BSC mainly after they quit anticancer therapies) was shorter in the sequential BSC group than the BSC alone group (36.0 days; 95% CI, 26.7 to 45.3 days vs. 98.0 days; 95% CI, 62.1 to 133.9 days; p < 0.0001) (Fig. 2b).

We conducted Cox regression analysis to elucidate the prognostic factors at the start of BSC in patients in the BSC group. The results of crude Cox regression analysis are shown in Table 3. Univariate analyses indicated 25 variables with p-values less than 0.1. The best model selected from 7 sets of models consisted of 10 variables; this best model was used for crude multivariate Cox regression analysis. Clinically significant poor prognostic factors were as follows: history of anticancer therapy, poor ECOG-PS, and presence of disseminated intravascular coagulation (DIC).

**Table 3:** Crude Cox regression analysis of survival from the beginning of best supportive care.

Variable	Univariate Analysis			Multivariate Analysis		
	HR	95% CI	p-value	HR	95% CI	p-value
Anticancer therapy (none vs. used)	1.40	1.26–1.55	0.0001	**1.73**	**1.27**–**2.37**	**0.001**
Performance status (0 → 4)	1.70	1.48–1.94	0.0001	**1.66**	**1.44**–**1.93**	**0.0001**
Disseminated intravascular coagulation (absent vs. present)	4.85	2.72–8.66	0.0001	**3.74**	**2.01**–**6.96**	**0.0001**
Age (low → high)	0.96	0.95–0.98	0.0001	0.97	0.95–0.98	**0.0001**
White blood cell (×10^3^) (low → high)	1.05	1.03–1.07	0.0001	1.02	1.01–1.04	**0.007**
Lactate dehydrogenase (×10^3^) (low → high)	1.01	1.003–1.02	0.007	1.01	1.00–1.02	**0.011**
Dementia (absent vs. present)	0.61	0.38–0.96	0.034	0.66	0.40–1.08	0.10
Alanine transaminase (low → high)	1.002	1.001–1.004	0.006	1.001	0.999–1.003	0.33
Brain metastasis (absent vs. present)	1.53	1.12–2.09	0.007	0.99	0.72–1.36	0.94
Blood urea nitrogen (low → high)	1.01	0.999–1.02	0.092	0.998	0.99–1.01	0.69
Stage (1 → 4)	1.30	1.14–1.48	0.0001		NI	
Hemoglobin (low → high)	0.84	0.79–0.89	0.0001		NI	
Albumin (low → high)	0.40	0.32–0.49	0.0001		NI	
C-reactive protein (low → high)	1.04	1.03–1.06	0.0001		NI	
Patient’s wish about best supportive care (absent vs. present)	0.45	0.32–0.62	0.0001		NI	
Doctor’s decision about best supportive care (absent vs. present)	2.13	1.48–3.09	0.0001		NI	
Transfer to hospice or home (absent vs. present)	0.44	0.37–0.53	0.0001		NI	
Liver dysfunction (absent vs. present)	1.46	0.96–2.21	0.074		NI	

Abbreviations: HR, Hazard ratio; C.I., confidence interval; Variables with a p-value < 0.10 on univariate analysis were entered into multivariate Cox analysis using.

the simultaneous method. NI, not included in the best multivariate Cox regression model.

Finally, PS-adjusted Cox regression analyses were carried out (Table 4). By PS-adjusted univariate analyses, 20 variables with p-values less than 0.1 were detected. The best model selected from among 13 models consisted of 10 variables. PS-adjusted multivariate Cox regression analysis revealed that poor prognostic factors from the start of BSC were poor ECOG-PS (hazard ratio [HR], 1.54; 95% CI, 1.31 to 1.82; p = 0.0001), presence of DIC (HR, 3.35; 95% CI, 1.75 to 6.39; p = 0.0001), and history of anticancer therapy (HR, 1.62; 95% CI, 1.17 to 2.24; p = 0.001).

**Table 4:** Propensity score adjusted Cox regression analysis of survival from the beginning of best supportive care.

Variable	Univariate analysis			Multivariate Analysis		
	HR	95% CI	p-value	HR	95% CI	p-value
Age (low → high)	0.96	0.95–0.98	0.0001	0.97	0.95–0.98	**0.0001**
Performance status (0 → 4)	1.62	1.40–1.88	0.0001	**1.54**	**1.31**–**1.82**	**0.0001**
Disseminated intravascular coagulation (absent vs. present)	4.38	2.40–8.00	0.0001	**3.35**	**1.75**–**6.39**	**0.0001**
Anticancer therapy (none vs. used)	2.07	1.51–2.84	0.0001	**1.62**	**1.17**–**2.24**	**0.001**
White blood cell (×10^3^) (low → high)	1.06	1.04–1.08	0.0001	1.03	1.01–1.05	**0.002**
Lactate dehydrogenase (×10^3^) (low → high)	1.01	1.001–1.02	0.025	1.01	1.002–1.02	**0.015**
Dementia (absent vs. present)	0.53	0.33–0.85	0.008	0.65	0.40–1.08	0.09
Alanine aminotransferase (low → high)	1.002	1.001–1.004	0.008	1.001	0.999–1.003	0.38
M factor (0 → 1)	1.33	0.99–1.78	0.061	1.09	0.77–1.55	0.62
T factor (1 → 4)	1.16	1.06–1.27	0.001	1.02	0.93–1.12	0.67
Hemoglobin (low → high)	0.87	0.82–0.93	0.0001		NI	
Albumin (low → high)	0.42	0.33–0.53	0.0001		NI	
C-reactive protein (low → high)	1.03	1.02–1.05	0.0001		NI	
Doctor’s decision about best supportive care (absent vs. present)	0.03	0.006–0.18	0.0001		NI	
Stage (1 → 4)	1.25	1.10–1.43	0.001		NI	
Number of chemotherapy courses (0 → 9)	1.08	1.01–1.16	0.018		NI	
Aspartic acid aminotransferase (low → high)	1.001	1.000–1.002	0.018		NI	
N factor (0 → 3)	1.13	0.995–1.28	0.060		NI	

Abbreviations: HR, Hazard ratio; C.I., confidence interval; Variables with a p-value < 0.10 on univariate analysis were entered into the multivariate Cox analysis.

using a simultaneous method. NI, not included in the best multivariate Cox regression model.

## Discussion

This study was conducted to investigate the decision-making factors for BSC alone and prognostic factors from the start of BSC in NSCLC patients. In this study, PS was used to adjust for differences in background characteristics. The decision-making factors for BSC alone by PS-adjusted multivariate logistic regression analysis were poor ECOG-PS, patients’ desire for BSC, wild type EGFR, presence of the relevant social background, and comorbidities such as dementia, pulmonary disease or psychiatric disorders. PS-adjusted multivariate Cox regression analysis revealed that poor prognostic factors from the start of BSC were poor ECOG-PS, presence of DIC, and history of anticancer therapy.

Due to severe adverse effects and long-term disabilities following cancer therapy, patients sometimes hesitate to receive anticancer therapies despite their being medically recommended. BSC alone is selected as the therapeutic option when the patient cannot tolerate aggressive anticancer therapies. ECOG-PS was a fundamental factor in both decision-making for BSC and prognosis from the start of BSC in this study. For NSCLC patients, ECOG-PS is one of the necessary key factors to decide the anticancer therapies including radical therapies or the systemic chemotherapies^13,14^. According to NCCN guideline and ESMO Clinical Practice Guidelines, NSCLC patients with poor performance status (especially ECOG-PS 3 to 4) are recommended not to receive aggressive anticancer therapies^14,15^. On the other hand, in patients harboring EGFR mutations, even patients with poor ECOG-PS experience the benefits of improvement in ECOG-PS and survival extension with the use of EGFR-tyrosine kinase inhibitors^16^, and hence, several guidelines have recommended administration of these agents in NSCLC patients irrespective of ECOG-PS. The results of this study that poor ECOG-PS and wild type EGFR are factors used in the selection of BSC alone are consistent with the descriptions in previous guidelines.

For patients with comorbidities, selection of BSC alone depends on the individual’s clinical situation. Patients with interstitial lung disease (ILD) have limited treatment options because aggressive anticancer therapies, such as surgery, radiation therapy, and chemotherapy, induce acute exacerbations^17^, radiation pneumonitis^18^, and drug induced ILD, which are sometimes fatal^19^. It is also difficult for patients with other pulmonary diseases, such as chronic pulmonary infection or severe chronic obstructive pulmonary disease, to receive aggressive anticancer therapies because of the worsening of infection or respiratory failure with these therapies.

Cancer patients with psychiatric disorders have higher mortality rates than the general population^20^. Several suggested causes for this are delays in the diagnosis and initial treatment of cancer, and the fact that the patients are less likely to receive standard treatment for cancer^21,22^. Several studies have also assessed cancer patients with dementia^23,24^. In the current study, BSC alone was selected for some patients with dementia or psychiatric disorders either by the patients themselves or by their clinicians if they were judged not to be suitable for aggressive anticancer therapy, although they had general indications for MTs.

Although median OS was longer in the sequential BSC group than in the BSC alone group, survival from the start of BSC alone was shorter in the sequential BSC group than the BSC alone group. PS-adjusted multivariate Cox regression analysis showed that patients receiving BSC after completion of anticancer treatment were 62% more likely to die than those receiving BSC alone. Continuing chemotherapy for advanced NSCLC for an extended interval leads to reduction in adequate supportive care such as symptom relief or hospice care and does not prolong survival^25,26^. Hence, oncologists should recommend discontinuation of anticancer therapy at an appropriate timing for improving patient QOL and prolonging survival. Further, consistent with a previous report^27^, the presence of DIC after the start of BSC was a poor prognostic factor in our study as well.

This study has several limitations. First, this was a retrospective study. Since the standard treatment for NSCLC is well established, a prospective observational cohort would be preferable. Although our study was a retrospective cohort study, we used PS-adjusted multivariate analysis to eliminate confounding factors as much as possible. As another approach, artificial intelligence such as a fuzzy and soft set theory or an ensemble model of an artificial neural network, which were used to predict postoperative mortality or morbidity after lung resection^28,29^, may be available to predict prognostic factors after the start of BSC alone. Second, because there has been remarkable progress in the treatment of NSCLC, such as the introduction of molecular targeted agents or immune checkpoint inhibitor antibodies^30^, the toxicities of these anticancer treatments are relatively reduced as compared to those of other kinds of treatment. Because of the duration of patients’ enrollment and follow-up period, no patients treated by ALK inhibitors or immune checkpoint inhibitor antibodies could be included in this study (Supplementary Table S4). Hence, it is possible that the indications for anticancer treatment might relatively broaden when the further study including the patients treated by newer chemotherapies will be conducted. Third, TNM staging in this study was classified according to not the latest 8th edition of the TNM Classification of Malignant Tumors^31^ but the 7th edition^10^. There are several changes including the subclassification of T and M factors and stage grouping between the 7th edition and the 8th edition. TNM staging is classified by an anatomical evaluation of tumor, and the treatment strategies have not changed dramatically. In this study, staging or T/N/M factors were not included in the best model or not significant in the multivariate analysis. We consider that the results in this report were sufficiently reasonable according to some published prognostic validation studies about 7th edition^32,33^; however, further research is necessary because there are several reports that the 8th edition is more effective in predicting prognosis^34,35^.

In conclusion, the decision-making process for BSC alone involves several diverse factors, such as cancer genetic factors, ECOG-PS, comorbid diseases, patients’ personal choices, and social background. When patients had poor ECOG-PS or occurrence of DIC at the time of decision-making regarding BSC, their survival duration was poor. In addition, the duration of BSC after completion of anticancer therapy was short because of an extended interval of chemotherapy. Hence, oncologists should recommend the proper withdrawal of anticancer therapy for improving QOL and prolonging survival in patients with NSCLC. Performance status is considered to be a pivotal factor in both decision-making and survival duration in patients receiving BSC.

## Supplementary information

Supplementary information

## Data Availability

The datasets generated during and analysed during the current study are available from the corresponding author on reasonable request.
