# Supplementary material for: Decision-making factors for best supportive care alone and prognostic factors after best supportive care in non-small cell lung cancer patients
Source: Sci Rep. 2019 Dec 27;9:19872. doi: 10.1038/s41598-019-56431-w (PMC6934749; doi:10.1038/s41598-019-56431-w)
Supplement: Supplementary file 1 — Supplementary information [file 41598_2019_56431_MOESM1_ESM.pdf]

Decision-making factors for best supportive care alone and prognostic factors after best supportive care in non-small cell lung cancer patients

Haruna Kitazawa, Yuichiro Takeda, Go Naka, Haruhito Sugiyama

Department of Respiratory Medicine, National Center for Global Health and Medicine

Supplementary Table S1 Background characteristics of the accrual group, n=354

	Best Supportive Care alone, n=118		Multidisciplinary therapy with supportive care, n=236	
Age, median (range)	78	(45-99)	69	(30-92)
Male (%)	88	(74.6)	160	(67.8)
Histologic type (%)				
Adenocarcinoma	62	(52.5)	177	(75.0)
Squamous cell	41	(34.8)	56	(23.7)
Others	15	(12.7)	3	(1.3)
Stage (%)				
IA/IB	3	(2.5)	43	(18.2)
IIA/IIB	9	(7.6)	16	(6.8)
IIIA/IIIB	26	(22.0)	59	(25.0)
IV	79	(67.0)	118	(50.0)
Unknown	1	(0.9)	0	(0.0)
Eastern Cooperative Oncology Group performance status (%)				
0	14	(11.9)	94	(39.8)
1	26	(22.0)	126	(53.4)
2	16	(13.6)	8	(3.4)
3	37	(31.4)	6	(2.5)
4	25	(21.1)	2	(0.9)
The data of diagnosis (%)				
~April 2009	45	(38.1)	120	(50.8)
May 2009~	73	(61.9)	116	(49.2)

	Best Supportive Care alone, n=118		Multidisciplinary therapy with supportive care, n=236	
Multiple comorbidities (%)	102	(86.4)	184	(78.0)
Pulmonary disease	60	(50.9)	78	(33.1)
Aspiration	16	(13.6)	3	(1.3)
Heart failure	28	(23.7)	50	(21.2)
Dementia	23	(19.5)	3	(1.3)
Renal dysfunction	20	(16.9)	18	(7.6)
Multiple cancers	20	(16.9)	41	(17.4)
Psychiatric disorders	9	(7.6)	5	(2.1)
Liver dysfunction	7	(5.9)	16	(6.8)
Hematological disorders	6	(5.1)	2	(0.9)
Disseminated intravascular coagulation	3	(2.5)	1	(0.4)
Public assistance / psychiatric hospitalization	15	(12.7)	11	(4.7)
Epidermal growth factor receptor mutation (%)				
Unknown	89	(75.4)	119	(50.4)
Wild type	25	(21.2)	65	(27.6)
Exon19 del	2	(1.7)	28	(11.9)
Exon21 L858R	2	(1.7)	18	(7.6)
Other type of mutation	0	(0)	6	(2.5)

Supplementary Table S2 Background characteristics of patients in the best supportive care group, n=278

	Best Supportive Care alone, n=118	Sequential Best Supportive Care, n=160
Age, median (range)	78 (45-99)	71 (40-94)
Male (%)	88 (74.6)	113 (70.6)
Histologic type (%)		
Adenocarcinoma	62 (52.5)	116 (72.5)
Squamous cell	41 (34.8)	40 (25.0)
Others	15 (12.7)	4 (2.5)
Stage (%)		
IA/IB	3 (2.5)	1 (0.6)
IIA/IIB	9 (7.6)	0 (0.0)
IIIA/IIIB	26 (22.0)	15 (9.4)
IV	79 (67.0)	144 (90.0)
Unknown	1 (0.8)	0 (0.0)
Eastern Cooperative Oncology Group performance status (%)		
0	14 (11.9)	1 (0.6)
1	26 (22.0)	18 (11.2)
2	16 (13.6)	19 (11.9)
3	37 (31.4)	72 (45.0)
4	25 (21.1)	50 (31.3)

	Best Supportive Care alone, n=118	Sequential Best Supportive Care, n=160
Multiple comorbidities (%)	102 (86.4)	155 (96.9)
Pulmonary disease	60 (50.8)	109 (68.1)
Aspiration	16 (13.6)	31 (19.4)
Heart failure	28 (23.7)	42 (26.3)
Dementia	23 (19.5)	8 (5.0)
Renal dysfunction	20 (16.9)	23 (14.4)
Multiple cancers	20 (16.9)	31 (19.4)
Psychiatric disorders	9 (7.6)	12 (7.5)
Liver dysfunction	7 (5.9)	23 (14.4)
Hematological disorders	6 (5.1)	3 (1.9)
Disseminated intravascular coagulation	3 (2.5)	10 (6.3)
Public assistance / psychiatric hospitalization	15 (12.7)	7 (4.4)
Epidermal growth factor receptor mutation (%)		
Unknown	89 (75.4)	83 (51.9)
Wild type	25 (21.2)	49 (30.6)
Exon19 del	2 (1.7)	16 (10.0)
Exon21 L858R	2 (1.7)	9 (5.6)
Other type of mutation	0 (0)	3 (1.9)

Supplementary Table S3 Initial treatment of Multidisciplinary therapy with supportive care and Sequential Best Supportive Care

	**Multidisciplinary therapy with supportive care, n=236**		**Sequential Best Supportive Care, n=160**	
surgery	42	17.8%	13	8.1%
Surgery + adjuvant chemotherapy or chemoradiotherapy	22	9.3%	6	3.8%
radiation therapy	20	8.5%	13	8.1%
chemoradiotherapy	22	9.3%	17	10.6%
chemotherapy	130	55.1%	111	69.4%

Supplementary Table S4 Regimen and line number of chemotherapy as an initial treatment

	Multidisciplinary therapy with supportive care, n=130		Sequential Best Supportive Care, n=111	
Regimen of 1st line chemotherapy				
Platinum doublet	85	65.4%	77	69.4%
Platinum doublet + Bevacizumab	10	7.7%	7	6.3%
Non-platinum doublet	1	0.8%	1	0.9%
Monotherapy	13	10.0%	12	10.8%
EGFR-TKI	21	16.2%	14	12.6%
The number of chemotherapy line				
1	40	30.8%	35	31.5%
2	28	21.5%	21	18.9%
3	30	23.1%	26	23.4%
4	12	9.2%	11	9.9%
5	7	5.4%	6	5.4%
6-9	13	10.0%	12	10.8%
The number of patients who was treated with EGFR-TKI after 2nd line chemotherapy	39	30.0%	33	29.7%

Abbreviation; EGFR-TKI, Epidermal Growth Factor Receptor- Tyrosine Kinase Inhibitor.
